# Association Between Preoperative Hydronephrosis and Prognosis After Radical Cystectomy Among Patients With Bladder Cancer: A Systemic Review and Meta-Analysis

**DOI:** 10.3389/fonc.2019.00158

**Published:** 2019-03-19

**Authors:** Jong Jin Oh, Seok-Soo Byun, Chang Wook Jeong, Cheol Kwak, Hyeon Hoe Kim, Ja Hyeon Ku

**Affiliations:** ^1^Department of Urology, Seoul National University College of Medicine, Seoul National University Bundang Hospital, Seongnam, South Korea; ^2^Department of Urology, Seoul National University College of Medicine, Seoul National University Hospital, Seoul, South Korea

**Keywords:** bladder cancer, hydronephrosis, stage, lymph node invasion, survival

## Abstract

**Background:** Preoperative hydronephrosis (HN) might be associated with adverse outcomes in patients who underwent radical cystectomy (RC). The aim of this study was to evaluate the effect of preoperative HN on the oncological outcomes in patients with bladder cancer who underwent RC by performing a systemic review and meta-analysis.

**Methods:** A systematic literature review in PubMed, EMBASE, and Scopus was conducted by searching the terms “bladder cancer,” “cystectomy,” and “hydronephrosis” until December 2016, in accordance with the Preferred Reporting Items for Systematic Review and Meta-analysis (PRISMA) guidelines. The calculated end points were advanced disease stage, cancer-specific survival (CSS), and overall survival (OS).

**Results:** Twenty-four studies involving 10,461 patients who underwent RC were included. Among the patients, 3,121 (29.8%) had preoperative HN. The pooled analysis showed that preoperative HN had a significant association with advanced stage (odds ratio, 2.56, 95% confidence interval [CI], 1.91–3.42, *p* < 0.00001), lymph node invasion (OR, 2.44, 95% CI, 1.79–3.34, *p* < 0.00001), CSS (hazard ratio [HR], 1.67, 95% CI, 1.34–2.08, *p* < 0.00001), and OS (HR, 1.51, 95% CI, 1.30–1.75, *p* < 0.00001).

**Conclusions:** Among patients with bladder cancer who underwent RC, preoperative HN could be a significant predictor of bladder cancer survival. However, large well-designed prospective studies are required to confirm the precise prognostic significance of preoperative HN.

## Introduction

Bladder cancer is the ninth most common cancer worldwide, with an estimated 430,000 new cases in 2012. More than 60% of all bladder cancer cases and half of all the 165,000 bladder cancer deaths occur in the less developed regions of the world (1). Radical cystectomy (RC) with pelvic lymph node dissection was regarded as the gold standard surgical method for muscle invasive bladder cancer (MIBC) and high-risk, non-MIBC (NMIBC) (2). Prediction of prognosis after RC was important for patient counseling, application of adjuvant treatment, and/or clinical trial. Several factors have been established to predict the prognosis after RC among patients with bladder cancer, such as pathological stage and lymph node invasion (3). However, another factor is needed to predict prognosis more accurately, therefore, several studies focused on identifying such a factor.

Preoperative HN is a common finding in patients with MIBC, with a reported incidence of up to 57.9% (4–6). Some researchers showed a positive association between prognosis and preoperative HN, and that preoperative HN had a significant association with extravesical disease, positive lymph node status, and cancer-specific survival (7, 8). However, other reports showed controversial results that preoperative HN was associated with higher stage but not with survival (3, 9). With preoperative imaging, prediction of prognosis was considered a relatively important step to decide appropriate treatment.

Therefore, in this study, we conducted a systemic review and meta-analysis of the available literature to obtain more-definitive results regarding the factor of preoperative HN during RC among patients with bladder cancer.

## Materials and Methods

A systematic review was performed in accordance with the Cochrane Collaboration and Preferred Reporting Items for Systematic Review and Meta-analysis (PRISMA) guidelines (10).

### Literature Search Strategy

A systematic literature search was performed in PubMed (1950–December 2016), EMBASE (1947–December 2016), and the Scopus Library using “bladder cancer,” “cystectomy,” and “hydronephrosis” as grouped terms.

### Overview of the Included Studies

**Figure 1:**
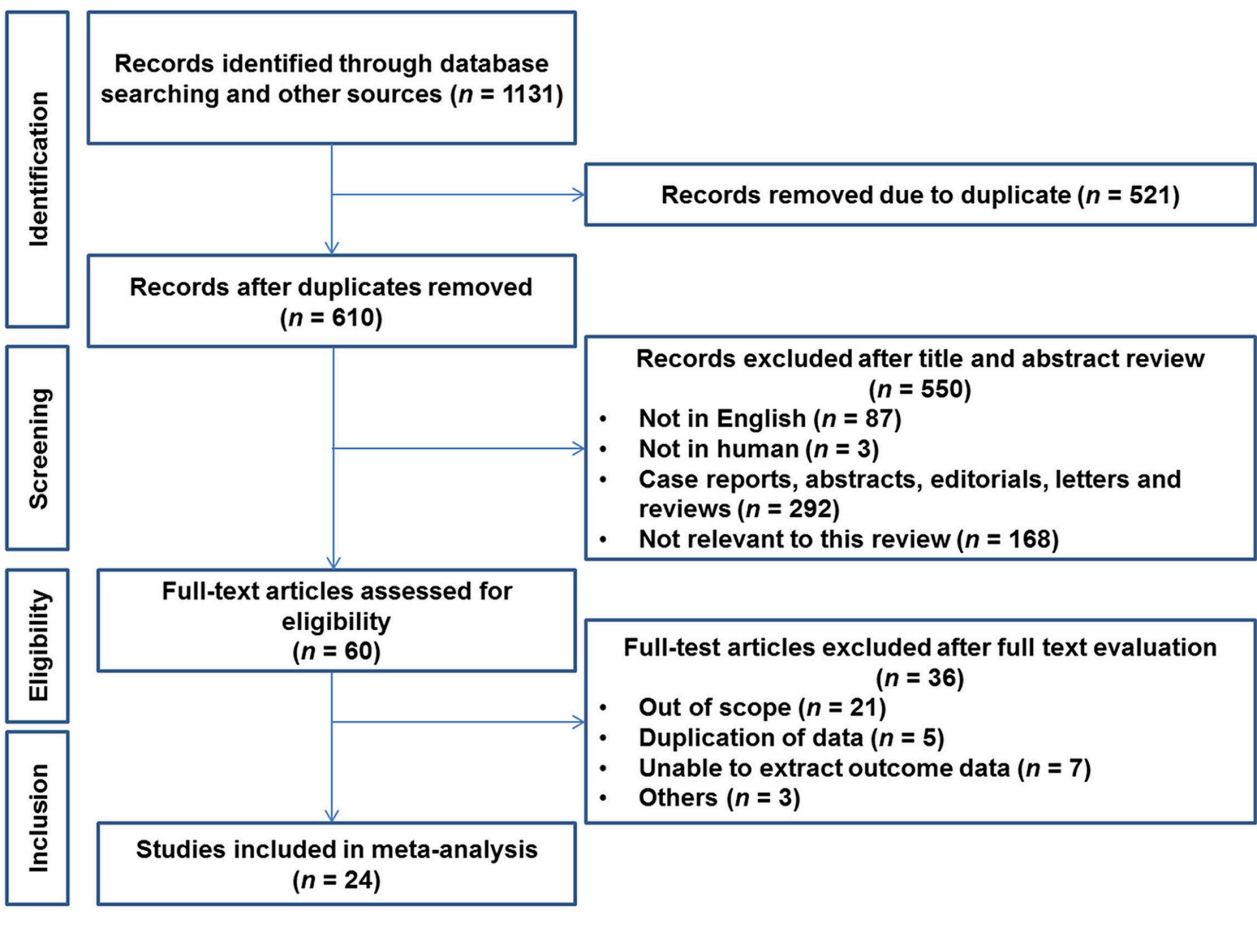
PRISMA statement flow diagram showing the search strategy for meta-analysis.

Figure 1 shows the PRISMA flowchart. We collected data from 237 PubMed articles, 380 Scopus articles, and 514 Embase articles, for a total of 1,131 resources. The exclusion criteria were duplicated data, non-English data, and non-human data. Finally, 24 studies we included in the meta-analysis (2–9, 11–26).

### Meta-Analysis

A meta-analysis was performed to summarize the gross prognostic value of associated factors. The cumulative effects of the factors were calculated using the inverse variance method. As traditional methods (27, 28), we measured the log odds ratio (OR) or hazard ratio (HR) and 95% confidence interval (CI) by using the indirect method. Statistical heterogeneity was investigated using the Cochran *Q-*test and the *I*^2^ statistic, which describes the percentage of total variation among the included studies. Significant heterogeneity was defined as a *p-*value of < 0.05 for the Cochran *Q-*test or an *I*^2^ statistic of >50% (29, 30). It resulted in the use of the random-effects model based on the Der Simonian method for estimating the tau value (29). To assess the risk of publication bias, we used a funnel plot and the Egger test for outcomes when at least 10 statistically significant studies were included in the meta-analysis (31). The meta-analysis was performed using the RevMan 5.0 statistical software (The Cochrane Collaboration, Copenhagen).

### Study Quality Evaluation

The study quality was determined by the Newcastle-Ottawa scale (NOS) for including cohort studies only (32). A total score of five or less was considered low, 6–7 was considered intermediate, and 8–9 high quality.

## Results

### Study Population

Table 1 shows individual data on the characteristics of the 24 studies and 10,461 patients population included. All the studies contained data from 46 to 1,776 patients selected according to the presence of HN. The proportion of patients without preoperative HN ranged from 45.7 to 83.1% in each cystectomy cohort. Among the 10,461 patients who underwent RC, 7,340 (70.2%) had no HN before RC and 3,121 (29.8%) had a hydronephrotic kidney.

**Table 1:** Characteristics of the studies included in the meta-analysis (studies = 24).

**No**.	**Study**	**Country**	**Recruited period**	**No. of patients**	**Median age**	**No HN (%)**	**Unilateral HN (%)**	**Bilateral HN (%)**	**Median fu (m)**
1	Canter	USA	1988–2003	306	65.3	232 (75.8)	57 (18.6)	17 (5.6)	45.6
2	Chapman	USA	1996–2006	308	66.4 (mean)	203 (65.9)	82 (26.6)	23 (7.5)	NA
3	Resorlu	Turkey	1990–2007	241	59.8 (mean)	189 (78.4)	39 (16.2)	13 (5.4)	34
4	Asadauskiene	Lithuania	2000–2008	46	60.5	21 (45.7)	24 (52.2)		18
5	Choudhury	UK	1995–2005	88	68	48 (54.5)	40 (45.5)		84
6	Kim	Korea	1986–2005	457	60.8	321 (79.1)	74 (18.2)	11 (2.7)	66.3
7	Stimson	USA	2001–2007	753	69	509 (67.6)	183 (24.3)	61 (8.1)	NA
8	Hofner	Germany	1990–2009	328	64	253 (77.2)	75 (22.8)		104
9	Lin	China	2003–2010	126	60	87 (69.0)	34 (27.0)	5 (4.0)	23
10	Xie	China	2003–2011	248	60	198 (79.8)	50 (20.2)		
11	Ahmadi	USA	1990–2008	1,186	66.9 (mean)	970 (81.8)	216 (18.2)		
12	Eisenberg	USA	1980–2008	1,776	68	1319 (74)	457 (26)		10.5 years
13	Green	USA		201	72.9	112 (72.3)	43 (27.7)		
14	Potretzke	USA	2002–2012	102	69.0	68 (66.7)	34 (33.3)		
15	Prelević	Serbia	2002–2012	233	63.8 (mean)	109 (46.8)	78 (33.5)	46 (19.7)	
16	Pietzak	USA	1990–2009	275		198 (72.0)	77 (28.0)		23.2
17	Racioppi	Italy	1982–2002	1,312	64.3 (mean)	751 (57.2)	176 (13.4)	40 (3.0)	39.0
18	Stojadinovic	Serbia	2002–2012	183	63.4 (mean)	77 (42.1)	106 (57.9)		
19	Hirasawa	Japan	2003–2015	136	68.6 (mean)	106 (77.9)	30 (22.1)		46.7
20	Martini	Europe	2011	337	69	258 (76.6)	79 (23.4)		
21	Mitra	USA	1971–2009	828		513 (62.0)	182 (38.0)		
22	Aglamis	Turkey		100	56.1 (mean)	64 (64.0)	36 (36.0)		27.7
23	Fernández	USA	1989–2012	103	67	79 (76.7)	22 (21.4)		60
24	Bartsch	Germany	1986–2003	788	65	655 (83.1)	108 (13.7)	25 (3.2)	35

*HN, hydronephrosis*.

### Locally Advanced Disease Prediction

**Figure 2:**
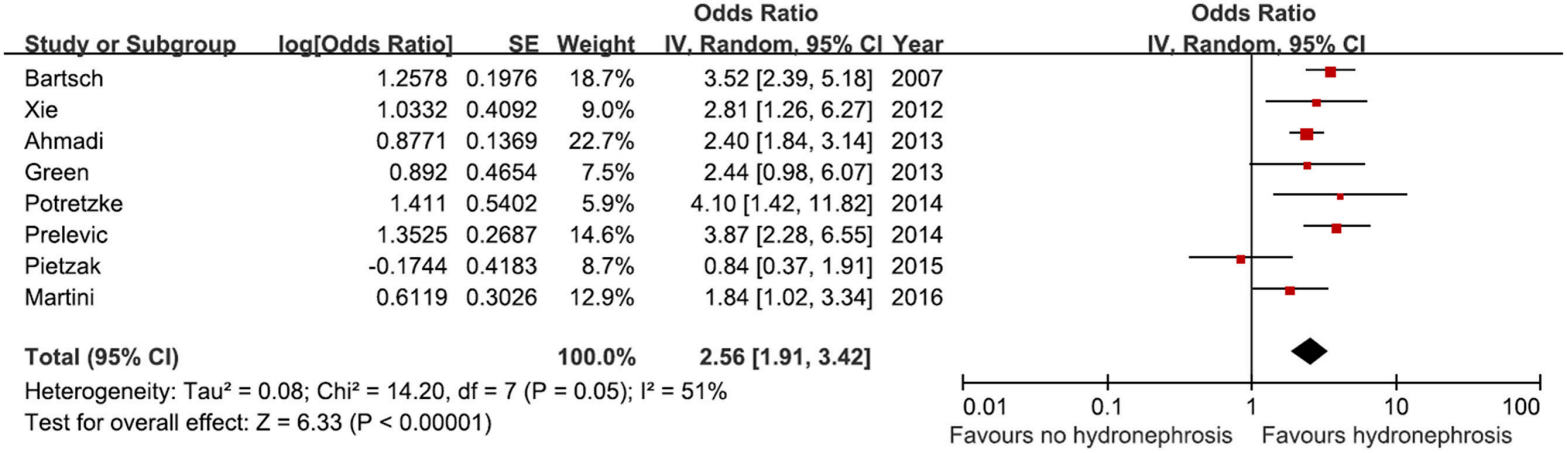
Forest plot and meta-analysis of advances pathological stage after radical cystectomy according to preoperative hydronephrosis. SE, standard error; IV, inverse variance; CI, confidence interval; df, degrees of freedom.

Eight studies involving 3,370 patients were identified for investigation of the prediction of locally advanced disease after RC according to the presence of preoperative HN. All the studies showed an odds ratio (OR) of 1.84 to 4.10 except one study (Figure 2). Among 3,370 patients, 802 (23.8%) had preoperative HN, the pooled analysis showed a significant difference in the prediction of locally advanced disease between the non-HN and HN groups (OR, 2.56, 95% CI, 1.91–3.42, *p* < 0.00001).

### Lymph Node Invasion

**Figure 3:**
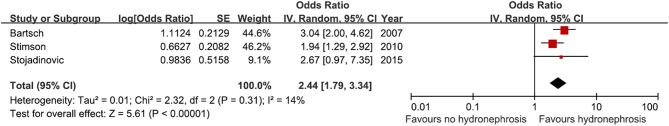
Forest plot and meta-analysis of lymph node invasion after radical cystectomy according to preoperative hydronephrosis. SE, standard error; IV, inverse variance; CI, confidence interval; df, degrees of freedom.

Data on the association between lymph node invasion and preoperative HN were available from three studies. The pooled analysis of these studies showed a significant association (OR, 2.44, 95% CI, 1.79–3.34, *p* < 0.00001, Figure 3).

### Cancer-Specific Survival

**Figure 4:**
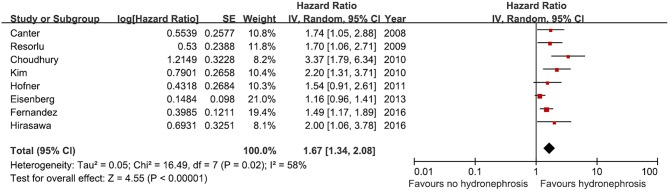
Forest plot and meta-analysis of cancer specific survival after radical cystectomy according to preoperative hydronephrosis. SE, standard error; IV, inverse variance; CI, confidence interval; df, degrees of freedom.

Eight studies were included in the meta-analysis to predict cancer-specific survival after RC. A total 3,435 patients were included, among whom 835 (24.3%) had preoperative HN and 2,600 (75.7%) had no preoperative HN. All the studies showed a positive association between preoperative HN and cancer-specific survival (HR, 1.16–3.37). A significant association was found in five studies but not in three studies (Figure 4). The analysis showed a significant association between bladder cancer-specific survival and preoperative HN among the selected cohorts (HR, 1.67, 95% CI, 1.34–2.08, *p* < 0.00001).

### Overall Survival

**Figure 5:**
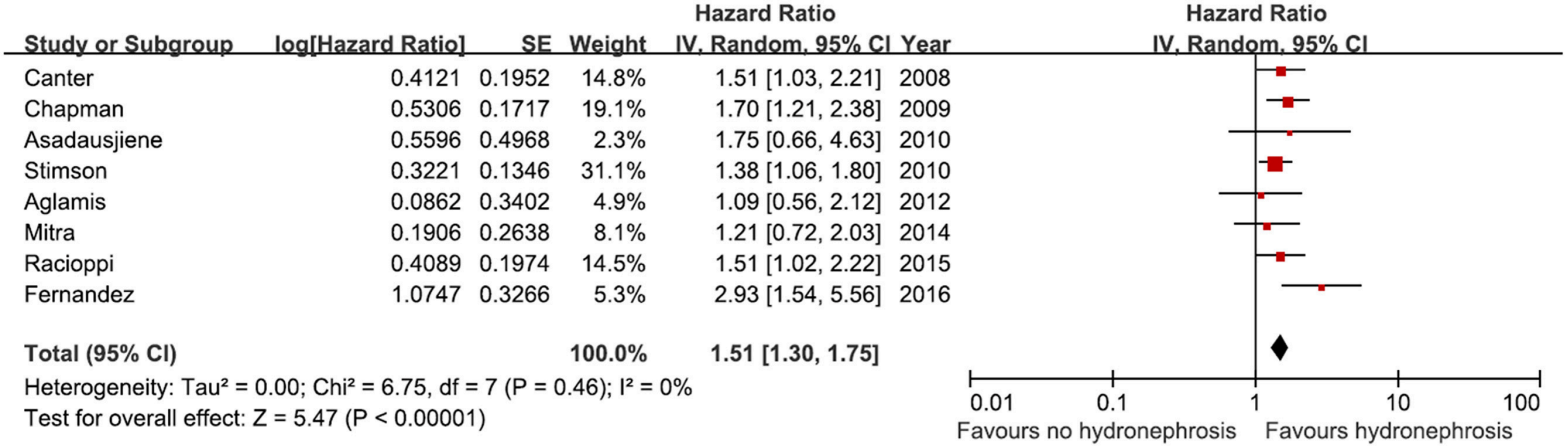
Forest plot and meta-analysis overall survival after radical cystectomy according to preoperative hydronephrosis. SE, standard error; IV, inverse variance; CI, confidence interval; df, degrees of freedom.

Eight studies were included in a meta-analysis to predict overall survival after RC according to preoperative HN. A total of 3,756 patients were included, among whom 903 (24.0%) had preoperative HN and 2,853 (76.0%) patients had no preoperative HN. From 2008 to 2016, all the included studies showed a positive association between preoperative HN and overall survival (HR, 1.09–2.93). A significant association was observed in five studies but not in three studies (Figure 5). The analysis showed a significant association between overall survival and preoperative HN among the selected cohorts (HR, 1.51, 95% CI, 1.30–1.75, *p* < 0.00001).

### Publication Bias

**Figure 6:**
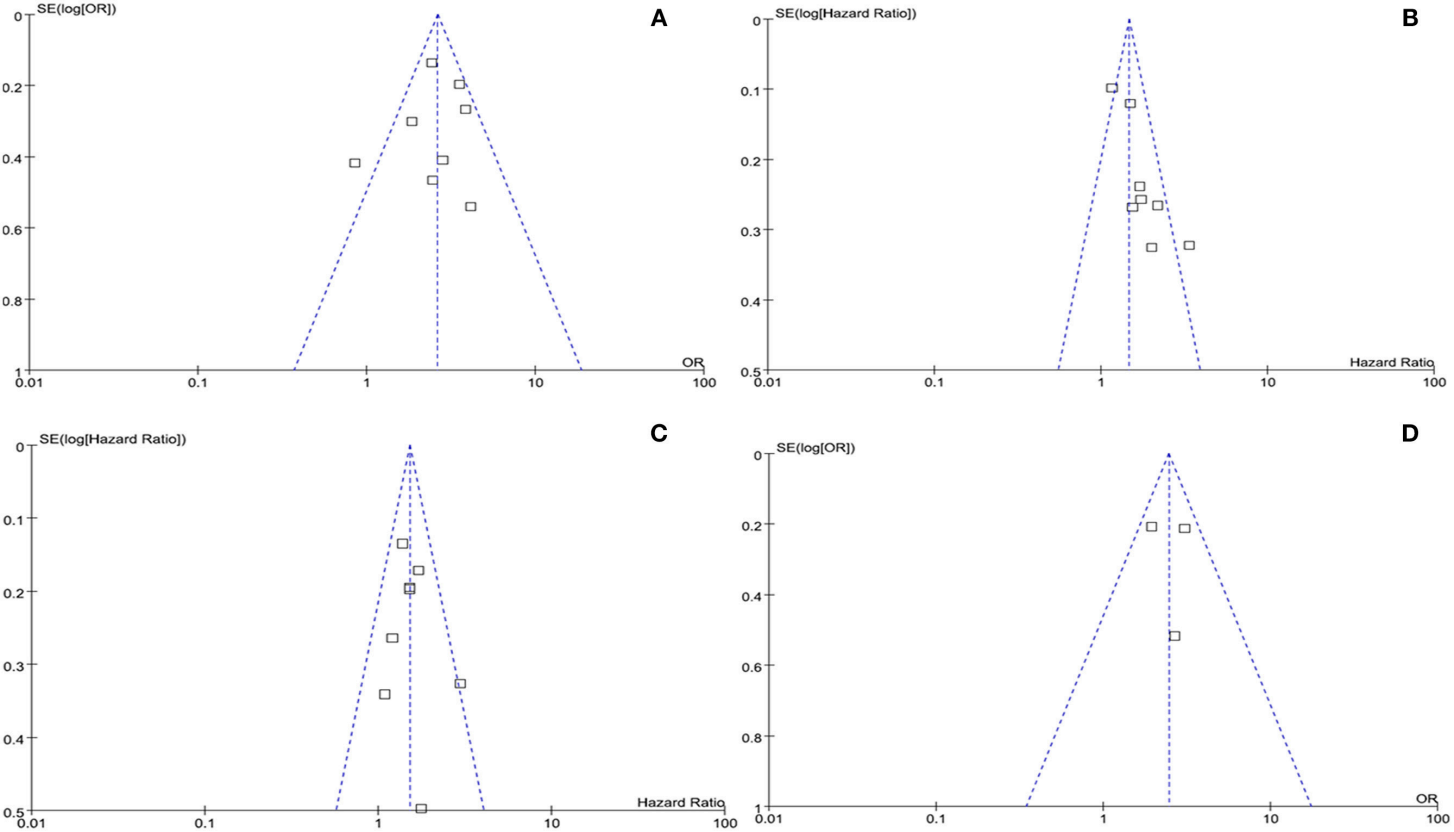
Funnel plots of **(A)** advanced pathological stage, **(B)** cancer specific survival, **(C)** overall survival. and **(D)** lymph node invasion. SE, standard error; OR, odds ratio.

The publication bias of our meta-analysis was assessed using funnel plots. No evidence of significant publication bias was observed (Figure 6). We found no strong evidence for publication bias by graphical inspection. No publication bias was detected in the other meta-analyses (all *p* > 0.05, Egger's test).

## Discussion

In this systematic review, we investigated the effect of preoperative HN on bladder cancer prognostic outcomes. A meta-analysis that incorporated 24 studies based on data from 10,461 patients who underwent RC for bladder cancer revealed that preoperative HN was significantly associated with pathologically advanced disease, lymph node invasion, cancer-specific survival, and overall survival.

Leibovitch et al. (33) evaluated 122 patients with invasive bladder cancer and showed for the first time the influence of HN on survival outcomes among patients who underwent RC. They showed ureteral obstruction (54.1%) due to bladder cancer associated with higher stage and grade, and low 5-year survival rate. Similar studies confirmed previous results that HN was an obvious prognostic factor. Thrasher et al. (34) reported their results from 531 patients who underwent RC for bladder cancer and demonstrated that preoperative HN was a significant prognostic factor of cancer-specific survival. Haleblian et al. (35) showed in their results in 1998 that 28% of patients with hydronephrotic bladder cancer had higher tumor stage and poorer survival outcomes.

On the basis of these results, preoperative HN could be a confirmatory factor for the prediction of patient survival after RC. However, some controversial results showed no association between HN and prognosis. Hofner et al. (3) reported their results on relatively long-term outcomes from an analysis of HN as a factor of cancer-specific survival. They showed that HN was positively associated with advanced stage, but not with cancer-specific survival. They emphasized the surgical margin status in a multivariate analysis, after including the aforementioned factors, HN remained a significant prognostic factor. Haleblian et al. (35) from the University of Southern California Group, reported a 5-year cancer-specific survival of 44.7% for patients with HN, which was lower than that of the patients without HN. However, cancer-specific survival was comparable after the follow-up period. More-large-scale data (*n* = 1,776) from the Mayo Clinic in the United States showed that preoperative HN was associated with a 20% increased risk of bladder cancer-related death. However, HN was not a significant factor in the multivariate analysis with a competing risk analysis (2). In our meta-analysis, eight studies were included in the analysis to examine cancer-specific survival. Six studies showed a positive impact on the increased HR, but two studies showed no significant impact. Finally, merged HR to cancer-specific survival was calculated to be 1.67 (95% CI, 1.34–2.08, *p* < 0.00001) in 3,435 patients with bladder cancer who underwent RC (2, 3, 7, 11, 13, 14, 22, 26). Overall survival was regarded controversial according to the presence of preoperative HN. In our meta-analysis, three studies showed no significant association between HN and overall survival (5, 24, 25), but five studies showed a significant association (8, 11, 12, 21, 26). The merged HR was 1.51 (95% CI 1.30–1.75, *p* < 0.00001) in 3,756 patients. As the prognosis of the patients with preoperative HN associated was so poor, more active therapeutic strategies might be beneficial for them (9).

The reason that the presence of preoperative HN had an impact on survival after RC might be the association with preoperative HN and pathological stage. Previous studies showed almost uniform results that HN before RC among patients with bladder cancer was a predictive factor of higher pathological stage. In the study by Leibovitch et al. (33), most cases of hydronephrotic bladder cancer without involvement of the intramucosal ureteral orifices extended to the external layer of the detrusor muscle. In their series, hydronephrotic bladder cancer infiltrating adjacent organs (pT4 tumors) was evident in 58% of patients. Therefore, preoperative HN was an evident factor of advanced disease stage and thus could be an apparent prognostic factor. Another reason should be focused on renal function. A previous study showed a significant cause-specific survival benefit for patients without HN as compared with those with HN (65.9 vs. 32.2% at 5 years) (4). The authors also found a significant difference in serum creatinine levels between patients with and patients without hydronephrotic kidneys (2.4 mg/dl vs. 1.1 mg/dl), and performed a simultaneous nephrectomy in 13.9% during surgical procedures. Therefore, decreased renal function could play an effective role in the prognosis after RC among patients with bladder cancer. Lastly, the appropriate treatment in the presence of HN might be limited to neoadjuvant chemotherapy because of the relative decrease in renal function (11).

This systemic meta-analysis has several limitations. First, most of the included studies had a retrospective design. No prospective study was included for comparison of outcomes according to preoperative HN, although we collected well-designed retrospective studies. Nevertheless, a prospective observational study should be conducted to confirm the significance of HN. Most included studies had an intermediate-to-high NOS score. Second, only few studies had a relatively small number of subjects included, and most of the studies had a relatively large sample size, therefore, publication bias analysis could include most of them. We also examined the differences between unilateral and bilateral HN as prognostic factors. However, only a relatively small number of studies compared unilateral HN to an extension on both kidneys: Nine studies included data for uni- and bilaterality, and only one study presented a statistically significant difference for CSS with a worse outcome among patients with bilateral HN compared to unilateral HN. Due to the lack of evidence no further analyses comparing unilateral and bilateral HN with respect to OS or CSS were possible. Another limitation was heterogeneity. The heterogeneity of studies were found in CSS. And no variables analyzed in the meta-regression contributed to the heterogeneity. In fact, the presence of heterogeneity may result from many other factors. Due to lack of detailed data, we could not use these variables in the meta-regression. Finally, we could not adjust the surgical methods, including urinary reconstruction. The obvious differences among urinary diversion techniques such as ileal conduit, neobladder, or ureterostomy could affect patients' health. However, we could not unify these factors. This could also be elucidated in a future prospective study.

In conclusions, our meta-analysis showed that patients with bladder cancer who had HN before RC had significantly higher pathological stage, higher risk of lymph node invasion, and poorer cancer-specific survival and overall survival than those who did not have HN before RC. Although larger prospective studies should be necessary to confirm our findings, preoperative HN was a confirmatory prognostic factor of survival in patients with bladder cancer.

## Author Contributions

JK conceived, designed, supervised experiments. CJ, S-SB, JK, HK, and CK performed the experiments. CJ and JK analyzed the data. JO wrote the manuscript.

### Conflict of Interest Statement

The authors declare that the research was conducted in the absence of any commercial or financial relationships that could be construed as a potential conflict of interest.
